# Artificial intelligence-based psychotherapy: focusing on common psychotherapeutic factors

**DOI:** 10.3389/fpsyt.2025.1710715

**Published:** 2025-11-11

**Authors:** Orestis Giotakos

**Affiliations:** Independent Researcher, Athens, Greece

**Keywords:** psychotherapy, AI psychotherapy, common factors, therapeutic alliance, eCBT, artificial intelligence–based psychotherapy

## Abstract

Artificial intelligence-based psychotherapy applications have been evolving rapidly in recent years. They seem to offer solutions to a complex world with enormous mental health needs. Easy access, immediacy and low cost are their enduring advantages, while anonymity attracts people who are isolated by the stigma of mental illness. Artificial intelligence-based psychotherapy applications borrow and incorporate elements of the already proven common psychotherapeutic factors, as described in the so-called ‘contextual model’. As decades of practice have shown, these ‘common factors’ seem to prevail in every type of in-person psychotherapy. They are the key elements of their successful outcome and the main reason for the lack of superiority of one type of psychotherapy over another. A key area here is therapeutic alliance, characterized by the therapist's empathy, the patient's expectations, and the shared therapeutic goals. Could artificial intelligence design opportunities so that these factors become even more useful in AI psychotherapy? Improvement in the development of an empathetic therapeutic relationship environment, based on the ‘theory of common factors’, are expected to facilitate the adaptation of interventions and further increase the reliability and effectiveness of AI psychotherapy.

## Introduction

As early as 1936, the American psychologist Saul Rosenzweig (1) observed that all types of psychotherapy seemed to be equally therapeutic, invoking the famous saying of the Dodo bird from the story "Alice in Wonderland": "Everybody has won and everybody should have prizes", to characterize the results of psychotherapy. He then proposed as a possible explanation some common therapeutic factors, including psychological interpretation, catharsis and therapist's personality. In 1940, John Watson reported the results of a scientific meeting held to determine areas of agreement between psychotherapeutic systems. Participants, including figures as diverse as Saul Rosenzweig, Alexandra Adler, Frederick Allen, and Carl Rogers, agreed that support, interpretation, insight, behavior change, a good therapeutic relationship, and certain therapist characteristics, were common features of successful psychotherapy approaches. (2).

After several decades of application, it has not been possible to prove that one psychotherapeutic approach is clearly superior to another. Specific psychotherapies such as Cognitive Behavioral Therapy (CBT), Mindfulness-based Cognitive Therapy (MCBT), Interpersonal Therapy (IPT), Acceptance and Commitment Therapy (ACT), and others, generally do not differ in effectiveness. This evidence suggests that factors common to all psychological therapies (i.e., “common factors”) contribute significantly to the therapeutic mechanism. (3–6). For example, the use of ‘transference’, a common element of the interpersonal relationship between patient and therapist, represents an important point of action in the psychotherapeutic process. It is an interesting phenomenon during psychotherapy, in which the patient redirects feelings or desires that were originally addressed to other people in his life onto the therapist. It is both a product of the psychotherapeutic encounter and a mechanism through which the therapy takes place. Could transference, or any other common psychotherapeutic factor, be constructed by programmers and engineers to design therapeutic methods based on artificial intelligence applications? More generally, how could artificial intelligence design opportunities where these factors are also useful in AI psychotherapies? (7).

We may suggest that the greatest integration of the common psychotherapeutic factors in AI-based psychotherapy will enhance the effectiveness and therefore the benefit the patients. The purpose of this article is to review the factors that are common to all types of in person psychotherapy, as a component of their successful outcome. Also, to detect these factors in Artificial Intelligence-based psychotherapy so far, through recent research, based on any use of artificial intelligence in psychotherapy, including AI-chatbots. Artificial intelligence (AI) is defined as the ability of a system to interpret external data, learn from it, and accomplish specific goals through adaptation (8). However, since the term ‘AI’ is used loosely, often simply to describe a classification model (9), the relative immaturity of the field is evident in the absence of consensus on the definition of AI and generative AI in the studies screened in this article.

## The common factors of successful psychotherapy

It is now widely accepted that the so-called ‘common factors’ contribute significantly to the success of psychotherapy. The common factors have a long history in the field of psychotherapy theory, research and practice and concern therapeutic alliance, empathy, expectations, cultural adaptation, and therapist differences. These factors are more than a set of therapeutic elements and are common to almost all psychotherapies that have been developed. Collectively, they form a theoretical model regarding the mechanisms of patient change during psychotherapy. The common factors model has been called the contextual model and argues that there are at least three mechanisms through which psychotherapy produces benefits: (a) the real relationship, (b) the creation of expectations, through the explanation of the disorder and the treatment involved, and (c) the implementation of health promoting actions. (10).

Before the work of psychotherapy begins, an initial bond must be created between therapist and patient, i.e. a therapeutic relationship. Some basic level of trust certainly characterizes all varieties of therapeutic relationships, although when attention is directed to more internal experiences, deeper bonds of trust and attachment are required and developed. The initial encounter between patient and therapist is essentially a meeting of two strangers, with the patient deciding whether the therapist is trustworthy, has the necessary experience to devote the time and effort to understanding both the problem and the context in which the patient and the problem find themselves. People make very quick judgments about whether they can trust their therapist. They make quick decisions based on the therapist's attire, the layout and decor of the office, and other features of the therapeutic environment. Patients also come to therapy with expectations about the nature of psychotherapy, based on past experiences, recommendations from close associates or influential individuals, and cultural beliefs. The initial interaction between patient and therapist is critical, as more patients appear to terminate therapy prematurely after the first session than at any other point during therapy (10–12).

The real relationship, as defined psychodynamically, is the personal relationship between therapist and patient, characterized by the degree to which each is genuine and perceives or experiences the other in ways that are appropriate to the other. The therapeutic relationship, or alliance, encompasses three central ideas: a collaborative relationship, an affective bond between the therapist and patient, and the ability of the therapist and patient to agree on treatment goals. Although the psychotherapeutic relationship is influenced by general social processes, the interaction is confidential, with some legal restrictions (e.g., reporting child abuse), and the disclosure of difficult material (e.g., spousal infidelity, etc.) does not disrupt the social bond. Indeed, in psychotherapy, the patient can talk about difficult material without the threat that the therapist will end the relationship. The importance of human connection has been discussed for decades, with concepts such as attachment, belongingness, or social support. Psychotherapy provides the patient with a human connection with an empathic and caring person, which promotes health, especially for patients who have poor or chaotic social relationships (10).

Research also shows that expectations have a strong influence on experience (13). Critical to the course of expectations is that patients believe that the provided explanation and the subsequent therapeutic actions will correct their problems. Consequently, the patient and therapist should agree on the goals of the treatment as well as the tasks, which are two critical components of the therapeutic alliance. The creation of expectations in psychotherapy depends on a convincing theoretical explanation, provided to the patient and accepted by him, as well as on therapeutic activities that are consistent with the explanation and that are believed to lead to control of his problems. A strong therapeutic alliance indicates that the patient accepts the treatment and cooperates with the therapist, creating confidence in the patient that the treatment will be successful. Empathy, a complex process through which an individual can be influenced and share the emotional state of another, is considered essential for cooperation, goal sharing and regulation of social interaction, while at the same time reinforcing the influence of expectations (14).

**Box 1** lists the common factors that have been recorded in literature from time to time and concern the pre-treatment period, the characteristics of the client and the therapist, the therapeutic relationship, the structure and development of psychotherapy (2).

Box 1 **Common factors in psychotherapy**

• Customer attributes: Positive expectation, hope or faith / Distressed or incongruent client / Patient actively seeks help / Mental illness perceptions.

• Pretherapy preparation: Expectation of therapeutic success / Perceptions of treatment or outcome / Expectation for length of treatment.

• Therapeutic relationship: Development of alliance or relationship / Engagement / Transference.

• Treatment structure: Use of techniques or rituals / Focus on inner world exploration of emotional issues / A healing setting / There is interaction / Communication (verbal and nonverbal) / Explanation of therapy and participants' roles.

• Psychotherapist characteristics: General positive descriptors / Cultivates hope and enhances expectancies / Warmth or positive regard / Empathic understanding / Socially sanctioned healer / Acceptance.

• Psychotherapy processes: Opportunity for catharsis or ventilation / Acquisition and practice of new behaviors / Provision of rationale / Foster insight or awareness / Emotional and interpersonal learning / Reality testing / Success and mastery experiences / Persuasion / Placebo effect / Identification with the therapist / Contingency management / Tension reduction / Desensitization / Education and information provision.

In a more general and theoretical basis, Bordin (15) suggested that a strong ‘alliance’ between a client and therapist is crucial for a successful outcome. ‘Therapeutic alliance’ is based on three key elements: agreement on goals, agreement on tasks, and the bond between them. Bordin argued that the alliance is a component of all therapies, although the specifics of the required alliance vary depending on the therapeutic approach. Furthermore, he added: “Strength, rather than the kind of working alliances, will prove to be the major factor in change achieved through psychotherapy”.

It is also worth mentioning the degree of follow-up, continuation, and completion of psychotherapy, as a parameter that indicates both client motivation and satisfaction. Premature termination of treatment hinders the effective delivery of mental health services across various settings, consumer populations, and treatment modalities. Dropout after the first session estimating at 50% across various settings. Attrition research is complicated by differing therapist and client perceptions of treatment or outcome. Therapists expect treatment to last significantly longer than do clients. Clients prematurely ending treatment may recognize a lack of improvement and believe that additional sessions will not be helpful, a fact often missed by therapists. External factors, such as difficulties in finding mental health services, greater distance travel, placement on a waiting list, and having a longer wait from intake to first treatment session have repeatedly been linked with treatment dropout. Higher rates of attrition were found for patients with more severe diagnoses and more complex diagnostic pictures (i.e., psychosis or Axis II comorbidity). The type of treatment a patient receives also influences rates of dropout. For example, treatments involving both medications and therapy have consistently shown lower rates of attrition than either medication or therapy alone. Demographics, environment, psychological need, perceptions of illness and mental health treatments influence engagement and retention in treatment. Perceptions of mental health are likely to also influence the utilization of services. Mental illness is often perceived in a negative way by many ethnic minority groups (16).

One of the earliest strategies for reducing dropout was based on the idea that preparing clients for what would happen in therapy would improve attendance and reduce early dropout. Pretherapy preparation, consisting of education about nature and process of therapy, offers clients an expectation of therapeutic success, dispels therapy misconceptions, and has been shown to improve client attendance. Thus, use of a brief pretherapy training video, motivational interview, or both could dispel many misconceptions and increase the likelihood of retention. Another factor influencing dropouts is the often-differing expectations about treatment duration. Expectation for length of treatment seems a critical factor to address in conducting effective treatment. Indeed, significant reductions in attrition may be seen if the duration of treatment is clearly articulated and adapted to be more in line with consumers’ actual use of services (17).

## Detecting common factors in AI-based psychotherapy

The application of AI to online mental health care is still in its infancy. However, the impact of AI is proving to be impressive. Such tools are increasingly being integrated into practice, offering virtual psychotherapy services, assisting with diagnosis, facilitating consultations, providing psychoeducation, and providing treatment options (18–20). Natural Language Processing (NLP) helps analyze patient language in conversations, chats, emails, and social media posts. It can detect patterns related to mental health issues, such as depression or anxiety, and is a vital component of chatbots (21, 22). Following the machine learning approach, chatbots extract content from user input using Natural Language Processing (NLP) and could learn from conversations. They consider the entire context of the dialogue, not just the current line, and do not require a pre-defined response for every possible user input. Often, Artificial Neural Networks (ANN) are used to implement chatbots. Retrieval-based models use a neural network to assign scores and select the most likely response from a set of responses. In contrast, generative models synthesize the response, typically using deep learning techniques (18).

The future of mental health care seems to involve a hybrid approach, combining the strengths of artificial intelligence and human therapists (23). AI-powered therapy chatbots offer virtual psychotherapy services and have shown promising results in reducing symptoms of depression and anxiety and helping to address mental health issues in various populations, including the elderly. An AI assessment tool was shown to be 89% accurate in identifying and classifying patients’ mental health disorders from just 28 questions, without human input (24). Graham et al. (25) stated characteristically: “As AI techniques continue to be refined and improved, it will be possible to help mental health practitioners re-define mental illnesses more objectively than currently done in the DSM-5, identify these illnesses at an earlier or prodromal stage when interventions may be more effective, and personalize treatments based on an individual’s unique characteristics”.

In a review of research on mental health chatbots, Li et al. (21) noted that chatbots have the potential to effectively alleviate psychological distress and even result in the creation of therapeutic relationships with AI. Recent studies have shown promising results for AI applications, including mental health monitoring, psychoeducation, suicide risk assessment and prediction, identification of predictors of mental illness, delivery of psychotherapy, therapist training, personalization of online mental health care, mental health triage and decision-making, and promotion of therapeutic engagement (26). A recent study found that ChatGPT (4.0) performance in facial emotion recognition is in line with human performance (27), while Hwang et al. (28) found that ChatGPT(4.0) can generate psychodynamic forms from a case history, while adding additional psychoanalytic material can improve the results. Types of psychotherapy applied by AI include Cognitive Behavioral Therapy (CBT), Acceptance & Commitment Therapy (ACT), Dialectical Behavior Therapy (DBT), Mindfulness-Based Cognitive Behavioral Therapy, and Supportive Psychotherapy. However, due to the novelty of this technology, there are several unanswered questions and issues, such as limitations in language interpretation, biases in interacting with patients from different backgrounds, as well as unanswered issues of ethics, patient safety, and health policy (9, 29).

Online mental health care has several advantages over its in-person counterpart, primarily due to the added privacy and ability to access healthcare from anywhere with an internet connection. Furthermore, studies show that despite some concerns about the strength of the therapeutic relationship, online mental health care has similar effectiveness to in-person options for managing mental illness. For example, a study by Alavi et al. (30) showed that an online cognitive behavioral therapy (eCBT) program for depression had similar effectiveness and dropout rates to its in-person counterpart, with a medium to large effect size on managing depressive symptoms. Regarding user satisfaction with online psychotherapy tools provided through Artificial Intelligence, several studies reported that their study tool was warmly received and considered useful and encouraging by approximately 60% to 90% of users. These studies highlighted the number of interactions with the tool, the feeling of empathy and understanding, and the appropriateness of the dialogue, as important positive factors determining treatment outcomes and satisfaction (31). Friesem (32) describes as following the characteristics of digital empathy: “digital empathy explores the ability to analyze and evaluate another’s internal state (empathy accuracy), have a sense of identity and agency (self-empathy), recognize, understand and predict other’s thoughts and emotions (cognitive empathy), feel what others feel (affective empathy), role play (imaginative empathy), and be compassionate to others (empathic concern) via digital media”.

Artificial intelligence enables more personalized and adaptive responses using multiple modes of interaction, such as text and voice. Monitoring treatment progress, assessing risk, personalizing the treatment experience, and training new therapists are challenges for online mental health delivery. However, especially in the case of fully self-guided online psychotherapy, the lack of monitoring, risk assessment, and personalization can put the patient at increased risk of dropping out of treatment or worsening psychiatric symptoms (33). Ewbank et al (34), 35) applied a deep learning approach to large patient datasets obtained from a variety of eCBT programs for mental health symptoms. They found that time spent on cognitive and behavioral techniques was associated with higher odds of improvement and treatment engagement. Although the authors acknowledge that some non-treatment-related content—such as informative greetings—can be important to the session, too much of it can be disruptive and ultimately detrimental. They also found that patient statements that indicated a desire or commitment to change were associated with increased odds of symptom improvement and therapeutic engagement.

More detailed exploration of AI psychotherapies sheds light on the complex internal structure of the psychotherapeutic process. Sperandeo et al. (36) evaluated the possibility of describing the complexity of therapeutic relationships using the methods of machine learning and complex networks. They concluded that the use of graphs is a valid tool for the analysis of both the psychotherapeutic sessions and the evolution of the care relationship over time. Also, numerous suggestions on the dynamics within the patient–therapist system emerged from the construction of a complex network useful for describing the trend of psychotherapy. Chen et al. (37) proposed a hierarchical framework to automatically evaluate the quality of an Enhanced CBT interaction (eCBT). The experimental results suggest that incorporating the local quality estimator leads to better segment representations and to consistent improvements for assessing the overall session quality. Chien et al. (38) categorized participants in an eCBT program for depression into five treatment engagement categories, considering treatment platform usage (i.e., time spent on the platform, access to sessions and treatment tools, and treatment session completion) and treatment disengagement rate. They found that lower platform usage was associated with lower symptom improvement rates, and higher platform usage and lower disengagement rates were associated with higher symptom reduction for depression and anxiety symptoms. Gonzalez Salas Duhne et al. (39), applied a supervised machine learning (ML) approach to analyze data from an in-person and an online eCBT program for depression and identified five common variables that could predict a higher likelihood of early dropout from eCBT: younger age, ethnic minority membership, lower socioeconomic status, medication use, and higher baseline severity of depressive symptoms.

**Box 2** lists some examples of common factors investigated in Artificial Intelligence-assisted Psychotherapy. For example, Schiepek et al. (42) suggested that common factors of psychotherapeutic change and psychological hypotheses on motivation, emotion regulation and information processing of the client’s functioning can be integrated into a comprehensive non-linear model of human change processes. Their model contributes to the development of an integrative conceptualization of psychotherapy, which is consistent with the state of scientific knowledge of common factors, as well as other psychological topics, such as motivation, emotion regulation and cognitive processing. Also, motivational interviewing has promise in increasing clients' commitment to and involvement with therapy (44). In a study by Hadar-Shoval et al. (45), the emotional awareness of progressive artificial intelligence adapted to the personality characteristics of individuals with borderline personality disorder and schizoid personality disorder was studied for therapeutic purposes. ChatGPT showed that it can demonstrate cognitive abilities, in terms of emotional richness and intensity, adapted to specific personality disorders. Several studies in the field of artificial intelligence-assisted psychotherapy, aimed at predicting treatment response, have identified demographic characteristics associated with more prosperous and less marginalized populations as predictors of better treatment response, highlighting this potential bias in the data. Education level is a frequently cited predictor of treatment response in patients participating in eCBT (40, 41).

Box 2 **Examples of common factors investigated in Aι Psychotherapy**

• *Customer attributes:* Psychological distress (21) / Personality characteristics (45) / Age, ethnic minority, socioeconomic status (39) / Education level (40, 59).

• *Pretherapy preparation:* Informative greetings - desire or commitment to change (34, 35)

• *Therapeutic relationship:* Evolution of the care relationship over time (36

• *Treatment structure:* Motivation - emotion regulation (42)

• *Psychotherapist’ characteristics:* Empathy and understanding (31) / Cost-effective - works continuously - does not wear out or get sick - interacts in different languages ​​ (43).

• *Psychotherapy processes:* Quality of interaction (37) / Time spent on the platform - completion of therapy sessions - dropout rates (38) / Early treatment dropout (30, 39)

The systematic review of Cruz-Gonzalez et al. (46) presented the application of AI in mental health in the domains of diagnosis, monitoring, and intervention. The authors found that the AI methods most frequently used were support vector machine and random forest for diagnosis, machine learning for monitoring, and AI chatbot for intervention. The AI chatbot Fido focuses on dialogue to recognize and modify cognitive biases using Socratic questioning. It identifies suicidal ideation, guiding users to emergency hotlines. It utilizes the ABC technique from CBT, provides psychoeducation on mental health, and offers gratitude practice exercises (47). The CBT chatbot Emohaa, rooted in CBT principles, uses interactive exercises, like automatic thoughts training and guided expressive writing, to address irrational thoughts and enhance mental well-being in healthy adults (n = 301) (19). Danieli et al. (48) found that mixed treatment with in-person and AI chatbot TEO components was most effective in reducing stress and anxiety in active workers (n = 60) over 55 with stress symptoms and mild-to moderate anxiety. In university students (n = 181) with anxiety and depression symptoms were evaluated the viability, acceptability, and potential impact of using Tess, an AI-based chatbot that delivers brief text conversations as comprehensive support for mental health. An electronic psychoeducation book on depression was used in a control group. The users express Tess was found effective in addressing anxiety but not depressive symptoms (49).

## Perspectives

The purpose of this article was to highlight the common psychotherapeutic factors as tools for further improving AI-based psychotherapies. As discussed above, the core of these factors is the so-called ‘therapeutic alliance’, as has been described by numerous psychotherapists. It even seems that AI-based psychotherapies have the potential to implement the Bordin’s ‘strength’ of alliances, since AI-based psychotherapies have the advantage of at least immediacy and availability.

Although AI does not replace therapists, many AI applications and tools show that they can provide a reasonable degree of therapeutic support (18). Artificial intelligence is increasingly being used in healthcare, supporting both mental health professionals in diagnosing and finding the best treatments, and mentally ill patients by providing information and psychotherapeutic interventions. Advanced technologies such as big language models, which became popular with the launch of ChatGPT in 2022, are being explored for their potential in mental health care to generate sophisticated responses and interactions, supporting the mental health of those in need (50). Artificial intelligence provides support to clients in an overstretched mental health system, bridging the gap where traditional services struggle to meet the growing demand for treatment (52). It is true that a chatbot providing therapy can make the mental health system more accessible and successful for people who hesitate to talk to a doctor because they feel uncomfortable revealing their feelings. In fact, in some cases, chatbots may be better suited to meet patients’ wishes than human doctors because they are not biased against patients, while patients are not biased against chatbots due to gender, age, or race. It’s possible that patients worried about social stigma would feel more comfortable asking an AI for help rather than a GP or a human psychotherapist (23, 43).

Chatbots also do not wear out or get sick. They are cost-effective and can operate continuously throughout the day, which is especially useful for people who may have health problems outside of their doctors’ working hours. Chatbots could become surrogate for nonmedical caregivers. They can also interact in different languages ​​to help respond to specific patient needs (43). Although online mental health care aims to promote greater accessibility to services, especially for those who live far from in-person services, these systemic barriers can limit the intended benefit of these interventions. Future studies should acknowledge this factor and support accessibility to services via internet-enabled devices and support technological literacy for marginalized communities (31).

A major obstruction is the lack of valid real-world databases required to feed data-intensive AI algorithms (33). Actually, although diagnostic and therapeutic issues are relatively settled in formal psychiatry, there is considerable confusion among the public. In the real world, there is notable misunderstanding of terminology and concepts of diagnoses and treatments (52). As a result, both accurate diagnosis and effective treatment of mental disorders remain unfulfilled goals (53).

Another obstacle is the low number of studies that have investigated the issues under discussion so far. Data related to eCBT, ACT, predicting treatment dropout, identifying useful treatment aspects, identifying predictors of symptom remission, matching patients to appropriate treatments, predicting treatment adherence, and predicting symptom remission, are extracted from very few studies. Thus, without a defined standard or guidelines for the study and application of AI tools in online mental health care, most research groups choose to study and develop their own AI tools, comparison interventions, outcome measures, and intervention designs. Also, in several of the available studies, there is no appropriate comparator or control group, which may directly affect the observed effect of AI interventions and tools reported in the literature. In summary, the factors that may affect the generalizability of conclusions in AI psychotherapy research are: gender distribution (most studies include women), ethnicity distribution (most studies are conducted in the US, Sweden, and the UK), race (the population mainly identifies as white), and the language of the articles (exclusion of studies not written in English) (31).

Several Large Language Models LMMs have “passed” the US medical licensing examination. However, passing a written medical examination with medical knowledge does not imply the provision of safe and effective clinical services. There is a discordance between what today’s models can do and what may be expected of them in real-world clinical workflows (54). The transition from a large language model used to answer medical questions to a tool that can be used by healthcare providers, administrators, and consumers will require significant additional research to ensure the safety, reliability, effectiveness, and privacy of the technology (54, 55). To support the credibility of studies using ML algorithms in the health sector in the future, WHO (56) suggests six key factors: Justification of the need to use ML, adequacy of data, description of the algorithm used, results including model accuracy and calibration, availability of programming code, and discussion of internal and external validation of the model.

**Box 3** provides some examples of such proposed factors and related research directions. Strengthening therapeutic alliance and an empathic therapeutic relationship, are important examples of common psychotherapeutic factors that will enhance the effectiveness of AI-psychotherapies. In fact, research into their specific components is expected to reveal hidden aspects of the therapist-patient relationship or even enrich our neuroscientific knowledge. For example, reviewing the literature to determine how the brain perceives human and artificial intelligence as a “presence”, in examples of social perception and decision-making, Harris (51) wondered how much and in what way the brain’s response to artificial intelligences would change as people gain more experience with them and become more integrated into human life.

Box 3 **Suggestions for further integrating common factors into AI Psychotherapy - Suggestions for future research**

• *Strengthening the empathic therapeutic relationship* (10, 32)

Positive expectations / Warmth or positive regard / Empathic understanding / Acceptance / Foster insight or awareness / Education and information provision

• *Strengthening therapeutic alliance – Strengthening the strength of working alliance* (15)

• *Strengthening research in existing fields*

Pretherapy preparation (34, 35) / Expectation of therapeutic success / Perceptions of treatment or outcome / Acquisition and practice of new behaviors / Rates of satisfaction and improvement / Rates of dropout (30)

• *Promoting new fields of research*

On the balancing the demographic characteristics of the samples (age, gender, educational level, economic status) (39)

On the detection and utilization of those common factors with the greatest therapeutic power (28)

On the identifying and analyzing individuals who preferred AI psychotherapy, due to stigma against mental illness or for economic reasons (23, 43)

On the monitoring treatment compliance and therapy dropouts – Identifying the causes (30, 38)

On the detection of possible new therapeutic factors emerging with AI psychotherapy (36)

Investigating how the brain perceives human and artificial intelligence as a ‘presence’ (51)

Ending up, the development of Artificial Intelligence in psychotherapy requires the use of state-of-the-art technologies to avoid over-reliance on algorithmic counseling and minimize errors. Blindly following algorithmic counsel can lead to unintended consequences, such as oversimplifying human complexity. While machine learning can provide valuable insights and support in psychotherapy settings, it is imperative to maintain a balanced approach that maintains human contact and recognizes the limitations of algorithmic decision-making (57). Continuous training and development for professionals in the field is recommended to ensure a balanced integration of technology and human expertise. Further applications of computational methods need to be identified to improve the results in AI psychotherapy. Further collaborations are needed to develop specific algorithms for different psychotherapies and patient groups (58). At the same time, the greatest possible integration of ‘common therapeutic factors’ into AI psychotherapies, by creating a 'therapeutic alliance environment', will further enhance their immediacy, reliability and effectiveness (59, 60).

## Conclusion

With nearly 100 years of psychotherapy practice, the main characteristics of both the patient and the therapist, the dynamics of the therapeutic relationship and the intermediate functions that serve the progress of the patient have already been studied extensively. The pre-treatment period, with the development of appropriate expectations and information, plays an important role, while the individual application of specific psychotherapeutic techniques can serve the needs of some special populations of patients. So far, the various AI psychotherapy programs use a variety of psychotherapeutic techniques, while incorporating various common psychotherapeutic factors. Immediacy and accessibility will always be the strong point of AI psychotherapy, in a world with complex relationships and enormous therapeutic needs. Easy analysis of AI psychotherapy data will now be able to provide more accurate explanations for client behavior during treatment, including dropout. In the future, balancing the demographic characteristics of the samples and improvements in the development of an empathetic therapeutic relationship environment, based on common psychotherapeutic factors, are expected to facilitate tailoring interventions and further increase the reliability and effectiveness of AI psychotherapy.

## Data Availability

The original contributions presented in the study are included in the article/supplementary material. Further inquiries can be directed to the corresponding author.
